# Supplementary material for: Spin-EPR-pair separation by conveyor-mode single electron shuttling in Si/SiGe
Source: Nat Commun. 2024 Feb 13;15:1325. doi: 10.1038/s41467-024-45583-7 (PMC10864332; doi:10.1038/s41467-024-45583-7)
Supplement: Supplementary file 1 — Supplementary Information [file 41467_2024_45583_MOESM1_ESM.pdf]

Supplementary Information

Tom Struck,[1,2] Mats Volmer,[1] Lino Visser,[1] Tobias Offermann,[1] Ran Xue,[1] Jhih-Sian Tu,[3] Stefan Trellenkamp,[3] Łukasz Cywiński,[4] Hendrik Bluhm,[1,2] and Lars R. Schreiber[1,2]

[1]*JARA-FIT Institute for Quantum Information, Forschungszentrum Jülich GmbH and RWTH Aachen University, Aachen, Germany*

[2]*ARQUE Systems GmbH, 52074 Aachen, Germany*

[3]*Helmholtz Nano Facility (HNF), Forschungszentrum Jülich, Jülich, Germany*

[4]*Institute of Physics, Polish Academy of Sciences, Warsaw, Poland*

SUPPLEMENTARY NOTE 1: MOTIONAL NARROWING OF DEPHASING DUE TO INTERACTION WITH NUCLEAR SPINS

After a single realisation of the experimental procedure of singlet initiation, separation of electrons, and shuttling of one of them forth by distance d and then back, the expectation value of singlet return probability oscillates as $\cos(2\pi\nu\tau)$, where the frequency $\nu(d)$ is given by Eq. (2) in the main text. We write it as $\nu(d) = \nu_{\Delta g}(d) + \nu_{\text{hf}}(d)$, and assume that the spatial variation of g-factor of the shuttled electron is due to frozen-in disorder. The contribution to frequency from the hyperfine interaction with the nuclear spins, $\nu_{\text{hf}}(d)$, is given by

$$h\nu_{\text{hf}}(d) = \frac{1}{d}\int_0^d \Delta E_{\text{hf}}(x)\text{d}x = \frac{1}{d}\int_0^d [E^L_{\text{hf}} - E^R_{\text{hf}}(x)]\text{d}x \ ,$$
$$= E^L_{\text{hf}} - \frac{1}{d}\int_0^d E^R_{\text{hf}}(x)\text{d}x \ , \tag{1}$$

where E^L_{hf} is the Overhauser field acting on electron in the L QD, while $E^E_{\text{hf}}(x)$ is the Overhauser field acting on the shuttled electron when the expectation value of its position along the channel is x. We have

$$E^L_{\text{hf}} = \mathcal{A}\int |\Psi_L(x' + |x_L|, y', z')|^2 I(x', y', z')\text{d}^3 r' \ , \tag{2}$$

where $\mathcal{A} \approx 2.15$ μeV [1] is the hyperfine coupling to ^{29}Si nuclei, $x_L < 0$ is the position of the L QD, $\Psi_L(x, y, z)$ is the envelope wavefunction of the static electron, and $I_z(x, y, z)$ is the nuclear spin polarization density along the z direction of the external magnetic field. Note that the envelope wavefunction should be normalized in the the following way [2]

$$\int |\Psi(x, y, z)|^2 \text{d}x\text{d}y\text{d}z = \nu_0 \ , \tag{3}$$

where $\nu_0 = a_0^3/4$ is the volume of the primitive unit cell (PUC) with $a_0 = 0.54$ nm being the lattice constant of Si. For the Overhauser splitting of the shuttled QD we have an analogous formula:

$$E^R_{\text{hf}}(x_R) = \mathcal{A}\int |\Psi_R(x' - x_R, y, z)|^2 I(x', y', z')\text{d}^3 r \ , \tag{4}$$

where x_R is the position of the R QD.

The nuclear spins fluctuate on the timescale of the whole experiment, but on the timescale of a single shuttling ($\lesssim 1$ μs) they can be considered to be static. The measured singlet

return probability signal is then given by

$$\langle \cos\left(2\pi[\nu_{\Delta g}(d)\tau + \nu_{\mathrm{hf}}(d)\tau]\right)\rangle = \cos[2\pi\nu_{\Delta g}(d)\tau] \langle \exp(-i2\pi\nu_{\mathrm{hf}}(d)\tau)\rangle \ , \tag{5}$$

where $\langle ... \rangle$ denotes an ensemble average over nuclear states in the L QD and along the channel through which the electron is shuttled. At temperatures used in experiment and in absence of dynamical nuclear polarization, the distribution of Overhauser fields experienced by the electron at any location x is a zero-mean Gaussian [3], and the $\langle \exp(-i\phi(d))\rangle$ term averages to $\exp(-\langle\phi^2(d)\rangle/2) = \exp[-(\tau/T_2^*)^2]$, where

$$\left(\frac{1}{T_2^*(d)}\right)^2 = 2\pi^2\langle\nu_{\mathrm{hf}}^2(d)\rangle \ , \tag{6}$$

in which

$$\langle\nu_{\mathrm{hf}}^2(d)\rangle = \frac{1}{h^2}\langle(E_{\mathrm{hf}}^L)^2\rangle + \frac{1}{h^2 d^2}\int_0^d\int_0^d \langle E_{\mathrm{hf}}^R(x)E_{\mathrm{hf}}^R(x')\rangle \mathrm{d}x\mathrm{d}x' \ , \tag{7}$$

where we have used the fact due to vanishing overlap between the wavefunctions of the static electron and the shuttled electron in the EPR pair (i.e. $|x_L|$ much larger than the spatial extent of both wavefunctions along x), the Overhauser fields experienced by them are uncorrelated. We can thus write

$$\left(\frac{1}{T_2^*(d)}\right)^2 = \left(\frac{1}{T_{2,L}^*}\right)^2 + \left(\frac{1}{T_{2,S}^*(d)}\right)^2 \tag{8}$$

where the dephasing time in the L QD is given by

$$T_{2,L}^* = \frac{h}{\sqrt{2}\pi}\frac{1}{\sqrt{\langle(E_{\mathrm{hf}}^L)^2\rangle}} = \frac{\hbar\sqrt{2}}{\sigma_L} \tag{9}$$

where σ_L is the standard deviation of the Overhauser splitting of the electron in the L QD, $\sigma_L = \sqrt{\langle(E_{\mathrm{hf}}^L)^2\rangle}$. In the same way we can write the contribution to dephasing time of the shuttled electron as $T_{2,S}^* = \hbar\sqrt{2}/\sigma_S(d)$, where

$$\begin{aligned}\sigma_S^2(d) &= \frac{1}{d^2}\int_0^d\int_0^d \langle E_{\mathrm{hf}}^R(x_R)E_{\mathrm{hf}}^R(x'_R)\rangle \mathrm{d}x_R\mathrm{d}x'_R \\ &= \frac{1}{d^2}\int_0^d\int_0^d C_{\mathrm{hf}}(x_R, x'_R)\mathrm{d}x_R\mathrm{d}x'_R\end{aligned} \tag{10}$$

and where we have defined the autocorrelation function of the Overhauser field along the channel, $C_{\mathrm{hf}}(x_R, x'_R)$

We assume that the shuttled electron envelope wavefunction $\Psi_R(x,y,z)$ has a fixed shape, i.e. the QD does not get deformed during the motion, and that is it separable in x,y,z coordinates. The autocorrelation of the Overhauser field along the channel that appears under the integrals in Eq. (10) is then given by

$$C_{\rm hf}(x_R,x'_R) = \mathcal{A}^2 \int \mathrm{d}^3r \int \mathrm{d}^3r' \langle I_z(x,y,z) I_z(x',y',z')\rangle$$
$$|\Psi_R(x-x_R,y,z)|^2 |\Psi_R(x'-x'_R,y',z')|^2. \tag{11}$$

As $\Psi(\mathbf{r})_R$ is an envelope function that is approximately constant on length scale of a_0 and smooth on larger scales, the nuclear polarization density can be treated as "spatial white noise", i.e. its autocorrelation function is

$$\langle I(x,y,z) I(x',y',z')\rangle = \langle I_z^2\rangle \delta(x-x')\delta(y-y')\delta(z-z') \tag{12}$$

and $\langle I_z^2\rangle = I(I+1)/3$ which is equal to 1/4 for $I=1/2$ pertinent to ^{29}Si nuclear spins. Using this we arrive at

$$C_{\rm hf}(x_R,x'_R) = \mathcal{A}^2 \langle I_z^2\rangle \int \mathrm{d}^3r |\Psi_R(x-x_R,y,z)|^2$$
$$|\Psi_R(x-x'_R,y,z)|^2$$
$$= \mathcal{A}^2 \langle I_z^2\rangle \int |\Psi_R(x,y,z)|^2 |\Psi_R(x+\Delta x,y,z)|^2 \mathrm{d}^3 r \ , \tag{13}$$

where we can see that this autocorrelation is in fact a function of $\Delta x \equiv x_R - x'_R$. When evaluated for $\Delta x = 0$ it gives simply σ_R^2, the standard deviation of the Overhauser field experienced by an electron with envelope wavefunction $\Psi_R(x,y,z)$.

Assuming that the confinement potential along x is harmonic, the x-dependent part of the wavefunction is $\Psi(x) \propto \exp(-x^2/2L^2)$, and we have

$$\int e^{-x^2/L^2} e^{-(x+\Delta x)^2/L^2} \mathrm{d}x \propto e^{-\Delta x^2/2L^2} \ , \tag{14}$$

from which we get

$$C_{\rm hf}(\Delta x) = C_{\rm hf}(0) e^{-\Delta x^2/2L^2} \equiv \sigma_R^2 e^{-\Delta x^2/2L^2} \ . \tag{15}$$

We see now that L is the autocorrelation length of nuclear-spin induced spatially random spin splitting experienced by the traveling electron. We thus expect an enhancement of $T^*_{2,S}$ time compared to the static QD value of $T^*_{2,R}$ due to motional narrowing when $d \gg L$ [4].

After plugging the above formula for $C_{\rm hf}(\Delta x)$ into Eq. (10) we obtain

$$\sigma_S^2(d) = \frac{\sigma_R^2}{d^2}\int_0^d\int_0^d e^{-(x-x'^2)/2L^2}\mathrm{d}x\mathrm{d}x' \ ,$$
$$= \sigma_R^2\Big[\frac{2L^2}{d^2}(e^{-d^2/2L^2}-1)+\frac{\sqrt{2\pi}L}{d}\mathrm{Erf}\left(\frac{d}{\sqrt{2}L}\right)\Big] \quad (16)$$

and the exact formula for $T_2^*(d)$ dephasing time of the EPR pair with one electron shuttled over distance d and back is

$$\left(\frac{1}{T_2^*(d)}\right)^2 = \frac{\sigma_L^2}{2\hbar^2}+\frac{\sigma_S^2(d)}{2\hbar^2} \ , \quad (17)$$

with $\sigma_S^2(d)$ given by Eq. (16). Note that the σ_R^2 prefactor in Eq. (16) is related to the dephasing time for spin in a static R QD by $T_{2,R}^* = \hbar\sqrt{2}/\sigma_R$.

By least-square fitting $T_2^*(d)$ data points in Fig. 2g of the main text with Eq. 16 and 17 (labeled as $f_2(d)$ in Fig. 2g) yields $T_{2,\rm R}^* = (560\pm 20)\,$ns, $T_{2,\rm L}^* = (1040\pm 50)\,$ns and $L = (8\pm 2)\,$nm. This results in $T_{2,\rm S}^*(280\,\mathrm{nm}) = (2130\pm 220)\,$ns. These values are close to the values fitted by the fitting function $f_1(d)$ discussed in the main text: $T_{2,\rm L}^* = (1110\pm 90)\,$ns, $T_{2,\rm R}^* = (520\pm 20)\,$ns and $T_{2,\rm S}^*(280\,\mathrm{nm}) = (2460\pm 310)\,$ns.

For $d \ll L$ we have

$$\sigma_R^2(d\ll L)\approx\sigma_R^2\left(1-\frac{d^2}{12L^2}\right) \ ,$$

while for $d \gg L$ we have

$$\sigma_R^2(d\gg L)\approx\sigma_R^2(\frac{L}{d}\sqrt{2\pi}-\frac{2L^2}{d^2}) \ .$$

In the $d \gg L$ regime we thus have

$$T_{2,S}^*(d\gg L)\approx\frac{T_{2,R}^*}{(2\pi)^{1/4}}\sqrt{\frac{d}{L}}\frac{1}{\sqrt{1-\frac{2L}{\sqrt{2\pi}d}}} \ ,$$
$$\approx\frac{T_{2,R}^*}{(2\pi)^{1/4}}\sqrt{\frac{d+\frac{2L}{\sqrt{2\pi}}}{L}} \ . \quad (18)$$

The d-dependence of the above formula is very similar to the dependence of the simple $\sqrt{(d+l_c)/l_c}$ expression used in the main text, explaining the good agreement between fits of $T_{2,R}^*$ and L or l_c to the results of measurements.

Finally, let us note that σ_R^2 given by Eq. (13) evaluated at $x=x'$ is

$$\sigma_R^2 = \frac{f\mathcal{A}^2}{2N} \quad \to \quad T_{2,R}^* = \frac{\hbar\sqrt{2N}}{\sqrt{f}\mathcal{A}}$$

where $N = \nu_0 / \int |\Psi_R(x,y,z)|^4 \mathrm{d}^3 r$ is the effective number of primitive unit cells encompassed by the wavefunction, f is the concentration of ^{29}Si (equal to 0.049 in the case of natural Si relevant here), and the factor of 2 comes from two Si atoms per primitive unit cell. If we assume that $\Psi_R(x,y,z) \propto e^{-x^2/2L_x^2} e^{-y^2/2L_y^2}$ and the z dependence is crudely approximated by a square function of width z_0, then using the normalization from Eq. (3), we obtain $N = 2\pi z_0 L_x L_y / \nu_0$. Assuming $L_y \approx 15\,\mathrm{nm}$ and $z_0 \approx 4\,\mathrm{nm}$, we estimate $T_2^* \approx 760\,\mathrm{ns}$ for $L_x = 8\,\mathrm{nm}$ and $T_2^* \approx 1040\,\mathrm{ns}$ for $L_x = 15\,\mathrm{nm}$ (left QD is not in the shuttle potential and thus much less confined), which are in reasonable agreement with $T_{2,\mathrm{R}}^*$ and $T_{2,\mathrm{L}}^*$, respectively.

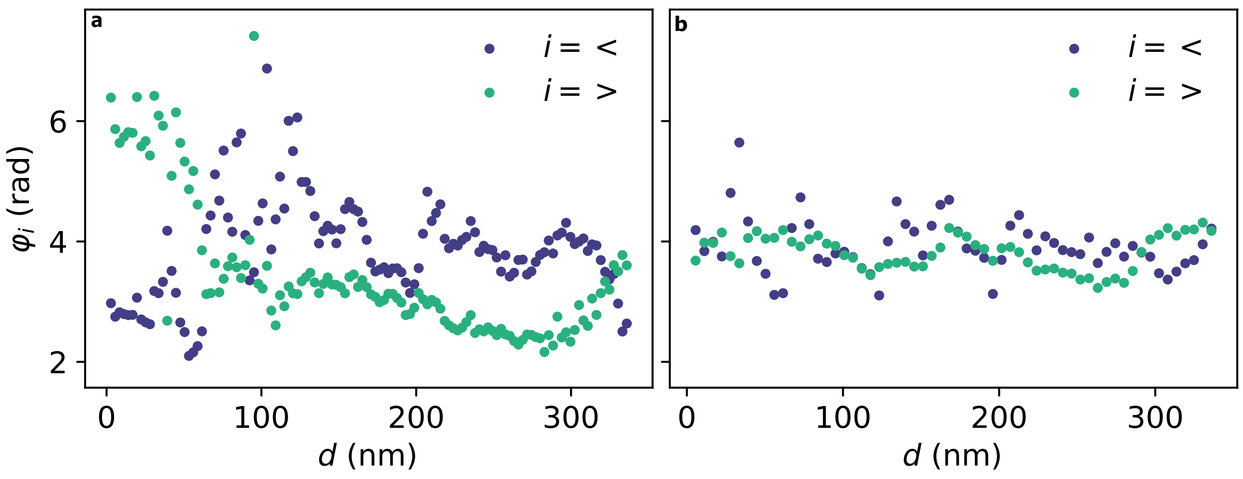

Supplementary Figure 1. Phases of the fitted oscillations. a: Data from the main text (Fig. 2d at $B = 0.6\,\mathrm{T}$). b: Data from the main text (Fig. 2c at $B = 0.8\,\mathrm{T}$).

SUPPLEMENTARY NOTE 2: EXTENDED DATA: PHASES OF THE FITS

In this section, we show the phase of the fitted oscillations from the main text (Fig. 2c,d). The data is shown in supplementary Fig. 1. Color-coding is the same as in the main text Fig. 2e and f. For a magnetic field of $B = 0.6\,\mathrm{T}$, the phases exhibit a significant spread at short distances. This might relate to the spread of the frequency ratio in the main text Fig. 2f at low distances. We suspect this might be due to fitting difficulties due to the small frequency difference between the low frequency branch and the large frequency branch, which is especially emanent for lower magnetic fields and small distances (main text Fig. 2e for $d < 120\,\mathrm{nm}$). The phase in Supplementary Fig. 1b is mostly constant.

SUPPLEMENTARY NOTE 3: EXTENDED DATA: LONG SHUTTLE DISTANCE FITS

In this section, we present a zoomed in version of the long shuttle distance fits (distance $D = 8, 10, 12\lambda$; main text Fig. 2h). Sinusoidal signal with the correct frequency is still observable proving partial entanglement.

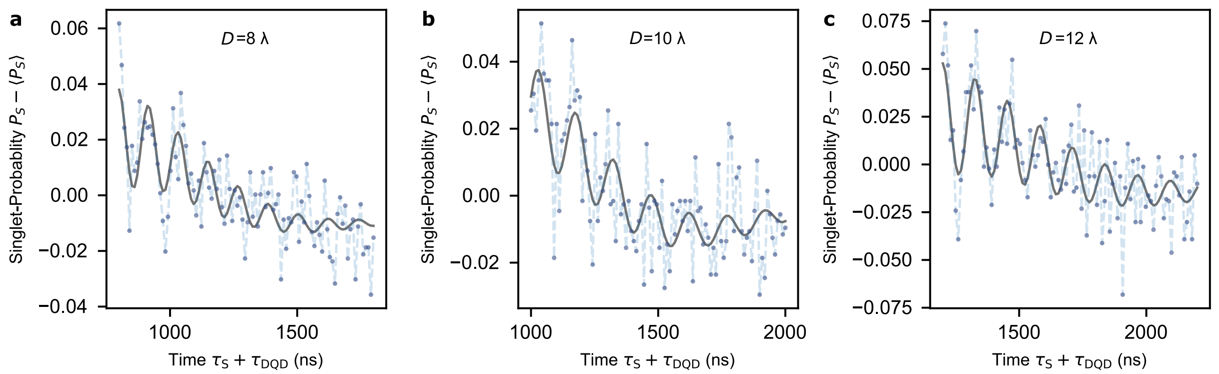

Supplementary Figure 2. Long distance coherent shuttling data with fits. These plots show a zoomed in version of the $D = 8, 10, 12\lambda$ from the main text Fig. 2h. The raw singlet-probabilities as a function of total time $\tau_S + \tau_{DQD}$ are indicated by the blue points with dashed lines as guides to the eye. The double sine fit as defined in the main text is given in grey. a: distance $D = 8\lambda$. b: distance $D = 10\lambda$. c: distance $D = 12\lambda$.

SUPPLEMENTARY NOTE 4: SHUTTLE PULSE OF THE CONVEYOR-MODE

In this section, we explain the conveyor-mode shuttle-pulses used in the experiment. We shuttle the electron into the 1DEC by applying sinusoidal pulses as explained in the main text.

$$V_{Si}(\tau_S) = U_i \cdot \sin(2\pi f \tau_S + \varphi_i) + C_i. \tag{19}$$

Then, we time-reverse the shuttle pulse voltages and return the electron to the DQD (see Supplementary Fig. 3). The time-reversal of the pulse translates to a direction flip on the travelling wave potential in the 1DEC that is induced by the shuttle pulse. The positions of the time reversals are shown in Fig. 3 by the dashed vertical lines for three different shuttle distances. These shuttle pulses are employed in order to record the data shown in Fig. 2 a-d of the main text. For main text Fig. 2 h, voltages are held constant for a duration τ_{DQD} after the shuttle pulse is performed with maximum shuttle velocity. For main text Fig. 3, the voltages are held constant for a time τ_W (at the vertical dashed line) instead of applying an immediate reverse pulse.

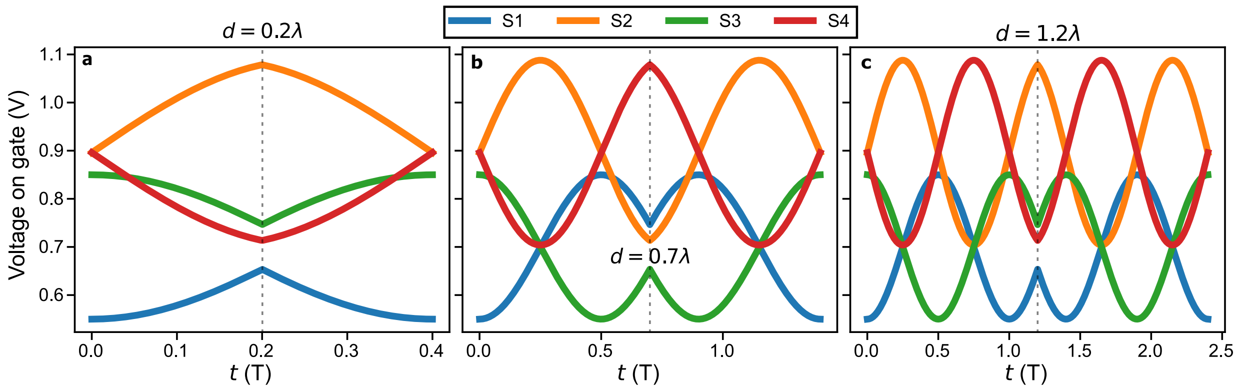

Supplementary Figure 3. Conveyor mode shuttle pulses used for obtaining the data in Fig. 2a,b of the main text. a: Shuttle pulse for a distance of $d = 0.2\lambda$. b: Shuttle pulse for a distance of $d = 0.7\lambda$. c: Shuttle pulse for a distance of $d = 1.2\lambda$.

SUPPLEMENTARY NOTE 5: STAGES OF THE POTENTIAL REACHED DURING PULSE SEQUENCE

In this section, we sketch the stages of the DQD potential in the experiment. In the separation stage S, the tunnel barrier under B2 is lowered and the DQD is detuned into the (3,1) regime by 20 mV on P1 (supplementary Fig. 4a). In the transport stage T, the tunnel barrier under B2 is pulled up by 130 mV lower voltage on B2 in order to secure the three electrons in the QD under P1 and avoid accidental tunnelling into an empty QD during shuttling (supplementary Fig. 4b). For the readout, the first pulse stage is the PSB stage P. Here, the barrier is again lowered and the DQD is tuned right into the PSB regime in order to allow only spin-singlets to tunnel (supplementary Fig. 4c). This area is given by the first orbital splitting E_{orb} in the left QD. After spin-to-charge conversion, the charge state is frozen against spin relaxation at pulse stage F. For freezing the charge state, we raise the tunnel barrier of the DQD by 130 mV lower voltage on B2 (supplementary Fig. 4d).

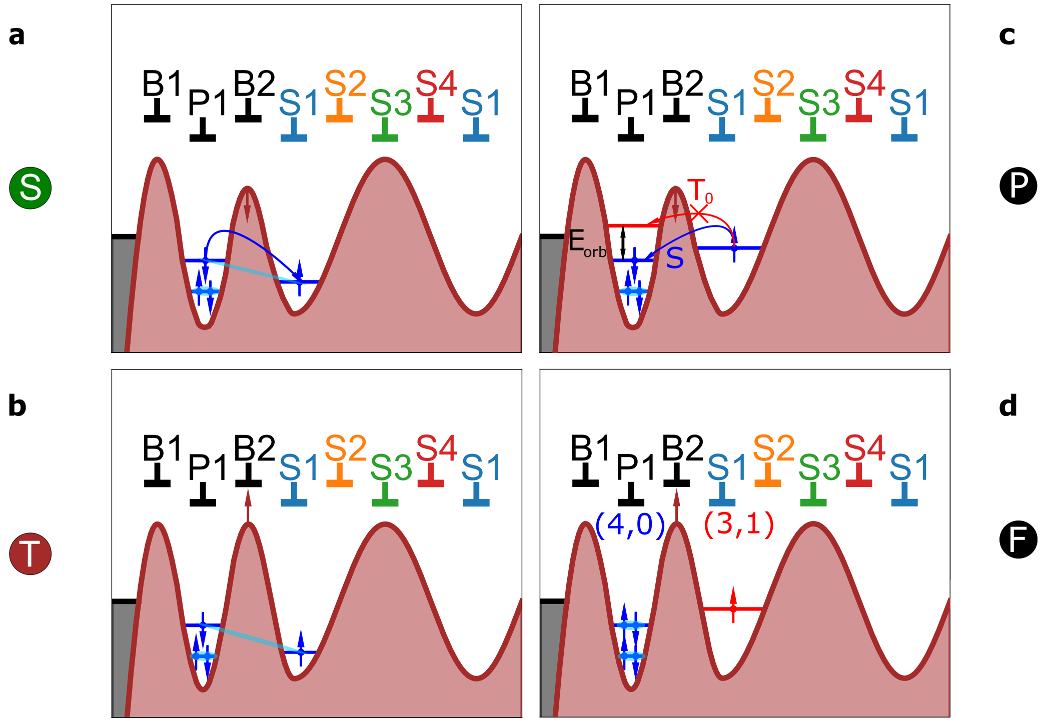

Supplementary Figure 4. Electrostatic potential sketch of the different pulse stages used in the experiment. a: Separation stage S. The tunnel barrier under B2 is lowered to allow one electron to tunnel to the shuttle QD under left S1. b: Transport stage T. Similar voltages to S, but the barrier under B2 is pinched off in order to avoid accidental tunnelling of electrons from the QD under P1 to the shuttle QD. c: PSB stage P. The tunnel barrier under B2 is lowered and the detuning of the DQD is put into the PSB regime. d: Freezing stage F. Similar voltages to P, but the tunnel barrier under B2 is pulled up. Either the charge state (4,0) (blue label for S-state) or (3,1) (red label for T_0 state) are detected.

[1] Assali, L. V. C. *et al.* Hyperfine interactions in silicon quantum dots. *Phys. Rev. B* **83**, 165301 (2011).

[2] Cywiński, Ł., Witzel, W. M. & Das Sarma, S. Pure quantum dephasing of a solid-state electron spin qubit in a large nuclear spin bath coupled by long-range hyperfine-mediated interaction.

Phys. Rev. B **79**, 245314 (2009).

[3] Merkulov, I. A., Efros, A. L. & Rosen, M. Electron spin relaxation by nuclei in semiconductor quantum dots. *Phys. Rev. B* **65**, 205309 (2002).

[4] Langrock, V. *et al.* Blueprint of a scalable spin qubit shuttle device for coherent mid-range qubit transfer in disordered Si/SiGe/SiO_2. *PRX Quantum* **4**, 020305 (2023).
